# Analysis of the characteristics of immune infiltration in endometrial carcinoma and its relationship with prognosis based on bioinformatics

**DOI:** 10.1097/MD.0000000000034156

**Published:** 2023-06-23

**Authors:** Yao Lin, Songyi Liu, Chunlin Lin, Penghang Lin, Zuhong Teng, Guangwei Zhu

**Affiliations:** a Department of Obstetrics and Gynecology, The First Hospital Affiliated to Fujian Medical University, Fuzhou, China; b Department of Gastrointestinal Surgery 2 Section, The First Hospital Affiliated to Fujian Medical University, Fuzhou, China.

**Keywords:** bioinformatics, endometrial carcinoma, immune, prognosis

## Abstract

To explore immune-related molecules that affect the prognosis of endometrial carcinoma (EC) using bioinformatic data mining. The expression data related to EC were downloaded from The Cancer Genome Atlas (TCGA) and Gene Expression Omnibus databases. After differential expression analysis, the intersection with immune related genes in the ImmPort database was used to obtain immune related differentially expressed genes (IRDEGs). The correlation between clinicopathological information and the prognosis of IRDEGs was further analyzed to obtain prognosis related differentially expressed immune genes (PRDEIG). Gene correlation analysis and Gene Set Enrichment Analysis (GSEA) enrichment analysis showed that PRDEIG was enriched in cancer-related functional pathways. We then analyzed the relationship between PRDEIG and immune cell infiltration, and further analyzed the mRNA and protein expression of PRDEIG in EC using TCGA and the human protein expression atlas (THPA) databases. After the intersection of the differential expression analysis results and immune-related genes, 4 IRDEGs were obtained: osteoglycin (OGN), LTBP4, CXCL12, and SPP1. After analyzing the relationship between 4 IRDEGs and clinicopathological parameters and prognosis of patients with EC, revealed that only OGN was not only related to tumor immunity, but also affected the prognosis of patients with EC. Gene correlation and GSEA enrichment of OGN were analyzed. The results showed that OGN was significantly enriched in 6 functional pathways: epithelial mesenchymal transition, KRAS signaling up, myogenesis, UV response, allograft rejection and apical junction. In addition, it was also found that OGN was significantly correlated with a variety of immune cells. The results of TCGA and THPA database showed that the mRNA and protein expression levels of OGN decreased in EC. OGN may affect the epithelial mesenchymal transformation (EMT) of tumor by affecting the infiltration of tumor immune cells.

## 1. Introduction

Endometrial carcinoma (EC) is the sixth most common cause of cancer death in women.^[1,2]^ In recent years, both incidence rate and mortality have shown an upward trend.^[3]^ At present, EC lacks specific markers, and its clinical diagnosis still depends on endometrial pathological biopsy.^[4]^ Furthermore, although surgical treatment improves the prognosis of early EC patients, the 5-year survival rate of EC patients with recurrence and metastasis is significantly lower.^[5]^ As targeted and immunotherapy develops, specific molecular markers have become more useful in improving cancer diagnosis and prognosis. Consequently, it is imperative to identify effective biomarkers for EC.

Individuals are burdened by cancer on many levels, including physical, mental, and economic. Through radiotherapy, chemotherapy, targeted therapies and immunotherapy, continuous efforts have been made to better understand tumor pathogenesis and improve treatment. The original intention of tumor immunotherapy is to restore the immune response of the body to the tumor.^[6,7]^ In addition, since the first Programmed Death 1/PD-L1 inhibitor was approved, many immune checkpoint inhibitors have also been used in clinic.^[8]^ In the field of immunotherapy, Cytotoxic T-lymphocyte antigen 4 and Programmed Death 1 immune checkpoints have marked a paradigm shift.^[9]^ Inhibitors of immune checkpoints target these molecules by alleviating certain inhibitory pathways, thereby promoting the immune system ability to combat tumors.^[10]^ In this regard, immune-checkpoint inhibitors are considered to be closely related to the host immune system as well as the tumor immune microenvironment in order to achieve their efficacy.

In the tumor immune response, the type of immune cell infiltration is crucial for the prognosis of tumor patients, because it determines whether the type of immune response tends to be anti-tumor or pro tumor.^[11]^ As a result, the development of new therapeutic strategies will depend on identifying new predictive biomarkers and understanding the tumor immune microenvironment in depth. However, there is still a lack of relevant research in EC. Therefore, this study is devoted to exploring the key genes of immune regulation related to EC and elucidating the key molecular mechanism of cancer.

In this study, The Cancer Genome Atlas (TCGA), Gene Expression Omnibus (GEO) and ImmPort databases were used to screen immune related differentially expressed genes (IRDEGs) in EC, and analyze their relationship with clinicopathological parameters and prognosis. To further analyze the functional pathways that prognosis related differentially expressed immune genes (PRDEIGs) may participate in and their correlation with immune cells, in order to provide a theoretical basis for the discovery of new immune targets in EC.

## 2. Materials & Methods

### 2.1. Data acquisition

All EC gene expression profile and clinical data were downloaded and sorted out from TCGA database (https://cancergenome.nih.gov/). Finally, 552 EC samples were included, of which 35 samples had adjacent paired tissues. From the GEO database, we found the expression profile data related to EC: GSE106191, including 64 EC and 34 normal tissue samples. ImmPort database (https//www.immport.org/home) was used to obtain immune related gene groups.

### 2.2. Screening IRDEGs

The differentially expressed genes (DEGs) of TCGA and GSE106191 datasets were corrected and analyzed by the “DESeq2” package of R software (V 4.0.4). The screening threshold of TCGA data was log2 fold change (FC) > | 3 | and adjP < 0.05. The filtering threshold of GSE106191 dataset was log2 FC > | 0.5 | and adjP < 0.05. The “ggplot2” package was used to visualize DEGs. In addition, IRDEGs were obtained by intersection of related genes through “VennDiagram” program package.

### 2.3. Analyzing the relationship between IRDEGs and clinicopathological parameters of EC

The clinical data in TCGA database were used to analyze the relationship between IRDEGs and clinical stage, histological classification and histological stage. The clinical stage of patients was classified into 2 groups, stage I to II and stage III to IV. In order to analyze the correlation between IRDEGs and clinical stage, IRDEGs expression differences in the 2 groups of patients were compared. A classification was also made based on the histological type of the patient, including endometrioid, mixed, and serous. Identifying if IRDEG expression is related to EC histological type by further analyzing patients with 3 different histological type. According to the histological stage of EC patients, they were classified into G1, G2, and G3 stage, and IRDEG expression was analyzed in 3 different histological stage to understand whether IRDEG affects it.

### 2.4. Analyzing the relationship between IRDEGs and prognosis of EC

The prognostic information in TCGA database was used to analyze the impact of IRDEGs on the prognosis of patients with EC, and then PRDEIGs was obtained. The median expression of IRDEG was taken as a starting point for categorizing all patients who had survival data into high and low expression groups. K-M survival curve analysis was also used to determine if IRDEG affects EC patients’ prognoses. PRDEIGs are genes in IRDEGs that can affect the prognosis of patients with EC.

### 2.5. Gene set enrichment analysis (GSEA)

GSEA is a computational method that can be used to analyze the potential functional pathways of a single gene. It was used to determine the enrichment of PRDEIG in different biological functions and signal pathways. The annotation gene set was selected as “h.all.v7.2.symbols.gmt,” and adjP < 0.05 was considered to be statistically significant.

### 2.6. Relationship between PRDEIGs and immune cell infiltration

The “GSVA” package of R software was used to analyze the relationship between PRDEIGs and 24 immune cells. Use the “ssGSEA” algorithm built into the “GSVA” package as the immune infiltration algorithm.

### 2.7. Analysis of mRNA and protein expression levels of PRDEIGs in EC

TCGA database was used to further analyze the differential expression of PRDEIGs mRNA in EC. The human protein expression atlas (THPA) (https://www.proteinatlas.org/) includes expression of proteins in cells and tissues.^[12]^ More than half of human protein coding genes are included in the project. THPA was used to analyze the protein level expression of PRDEIG in EC tissues and normal tissues.

### 2.8. Statistical analysis

Wilcoxon tests are used to compare continuous variables, which are summarized by mean and standard deviation. Analysis of variance compares categorical variables according to frequency and proportion. Log rank test for survival analysis. A number of packages related to the R software (version 4.0.4) were used to process, analyze, and present data. *P* < .05 on both sides was considered valuable.

## 3. Results

### 3.1. Acquisition of IRDEGs

**Figure 1.**
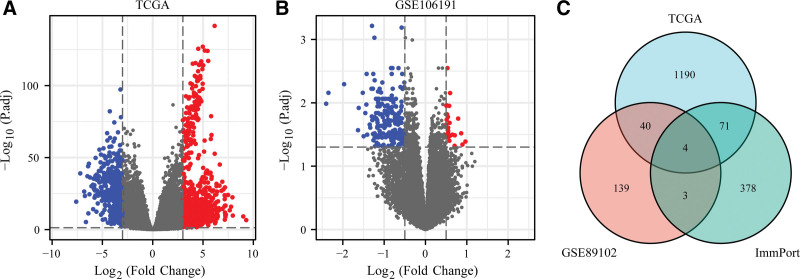
Screening of IRDEGs. (A) DEGs in TCGA database. (B) DEGs in GSE106191 dataset. (C) The intersection of DEGs in TCGA and GSE106191 datasets and immune related genes in ImmPort database to obtain IRDEGs. DEGs = differentially expressed genes, IRDEGs = immune related differentially expressed genes, TCGA = The Cancer Genome Atlas.

The differential expression between 552 EC samples and 35 adjacent tissues in TCGA database was analyzed. After screening according to log2 FC and adjP values, 1305 differentially expressed mRNAs were finally obtained, including 858 up-regulated and 447 down-regulated DEGs (Fig. 1A). Similarly, according to the threshold screening, 240 DEGs were obtained from the GSE106191 dataset, of which 26 were up-regulated and 214 were down-regulated (Fig. 1B).

The 2 groups of differentially expressed mRNA obtained above were intersected with the immune related gene groups obtained from the ImmPort database to obtain 4 IRDEGs (Fig. 1C), which are osteoglycin (OGN), LTBP4, CXCL12 and SPP1 respectively. There are 4 genes that are considered to be potential immune-related genes that may affect the occurrence and development of EC.

### 3.2. IRDEGs was correlated with clinicopathological parameters of EC

**Figure 2.**
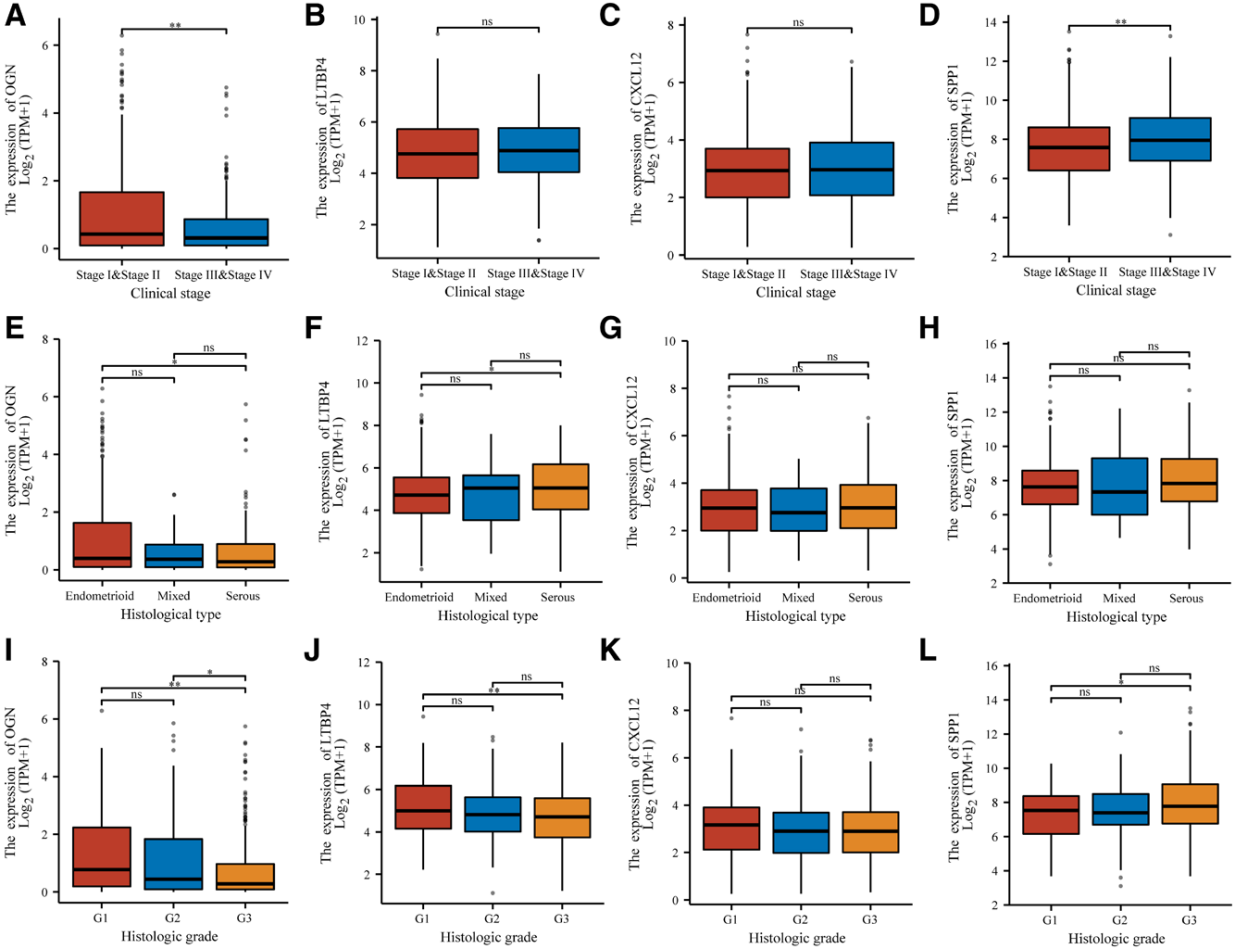
Relationship between IRDEGs and clinicopathological parameters of EC. (A) The OGN correlates negatively with clinical stage. (B and C) LTBP4 and CXCL12 are not associated with clinical stage. (D) SPP1 is positively correlated with clinical stage. (E and F) OGN and LTBP4 have significant correlations with histological type. (G and H) CXCL12 and SPP1 are not related to histological type. (I and J) Lower OGN and LTBP4 expression indicates a higher histological stage. (K) Expression of CXCL12 is not correlated with histological grade. (L) An increase in SPP1 expression indicates a higher histological staging. EC = endometrial carcinoma, IRDEGs = immune related differentially expressed genes, OGN = osteoglycin.

By analyzing the relationship between IRDEGs and clinicopathological parameters of EC, it was found that OGN was significantly correlated with clinicopathological stage, histological type and histological stage; LTBP4 was significantly correlated with histological classification and histological stage, but not with clinicopathological stage; CXCL12 is not related to any of the 3; SPP1 was significantly correlated with clinical pathological stage and histological stage, but not with histological type (Fig. 2). This leads us to speculate that downregulation of OGN expression may contribute more to EC malignancy.

### 3.3. IRDEGs affect the prognosis of EC

**Figure 3.**
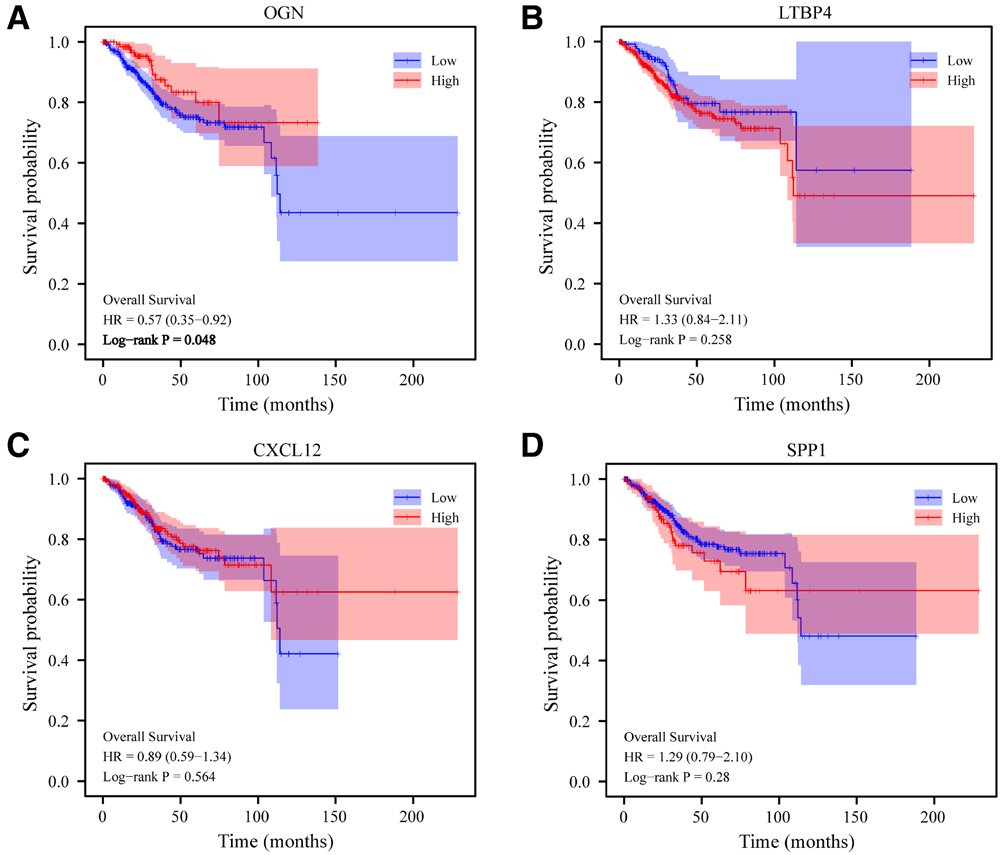
Relationship between IREDGs and prognosis of EC patients. (A) There is a better prognosis for EC patients with a higher expression of OGN. (B–D) A patient prognosis is not affected by the expression of LTBP4, CXCL17, and SPP1. EC = endometrial carcinoma, IRDEGs = immune related differentially expressed genes, OGN = osteoglycin.

To further analyze whether IRDEGs will affect the prognosis of EC. Kaplan–Meier survival analysis showed that only OGN could affect the prognosis of EC patients. The higher the expression of OGN, the better the prognosis of patients (Fig. 3A). There was no significant correlation between LTBP4, CXCL12 and SPP1 and the prognosis of EC patients (Fig. 3B–D). A clear link has been established between the downregulation of OGN expression and malignant progression of EC, as well as a potential prognostic molecule for the disease.

### 3.4. GSEA enrichment analysis

**Figure 4.**
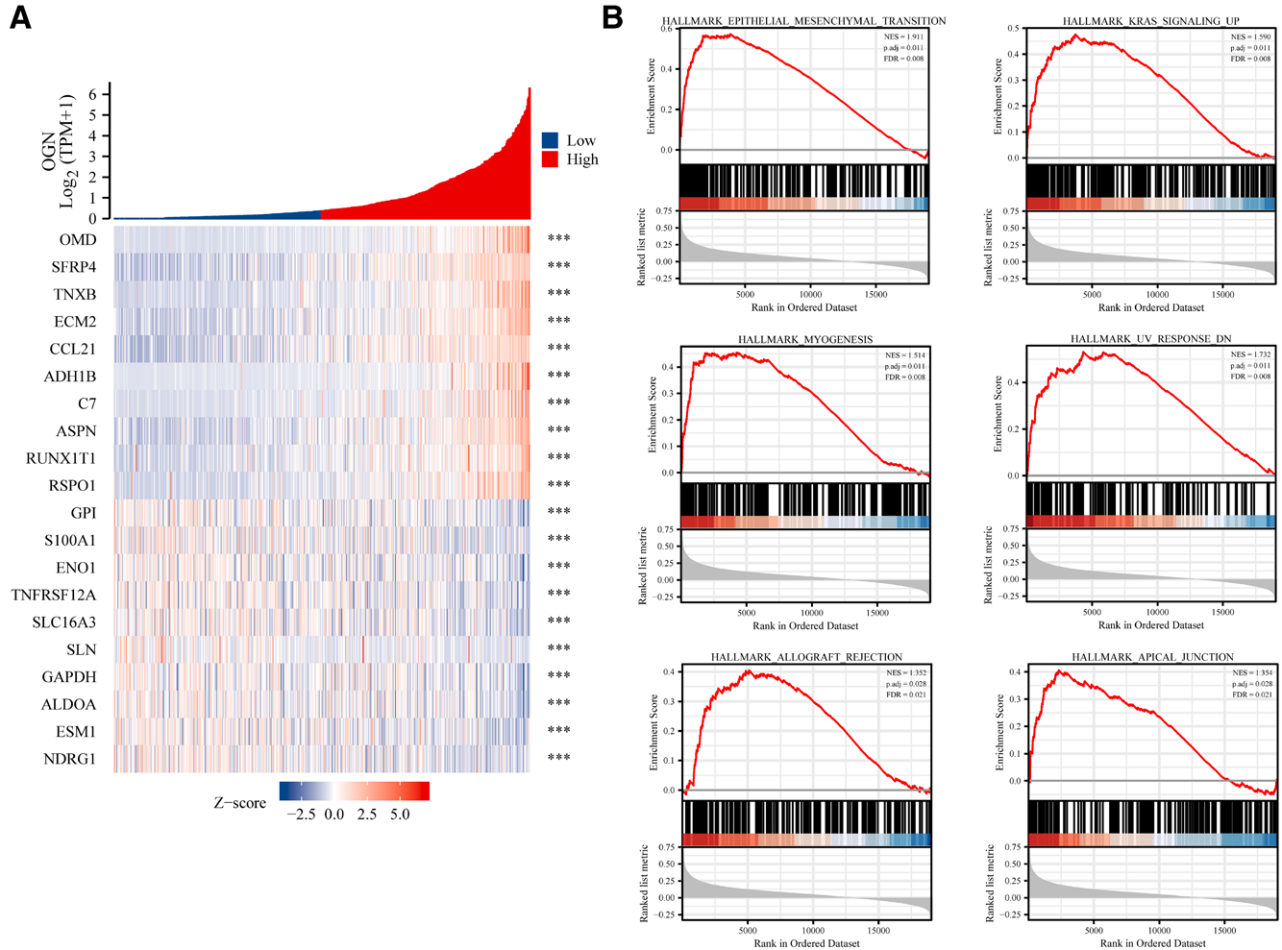
Gene correlation analysis of OGN and enrichment analysis of GSEA. (A) These are the top 10 genes with a positive or negative correlation with OGN expression. (B) OGN was significantly correlated with the following 6 functional pathways, namely EMT, KRAS signaling up, myogenesis, UV response DN, allograft rejection and apical junction, based on GSEA enrichment analysis. EMT = epithelial mesenchymal transformation, GSEA = Gene Set Enrichment Analysis, OGN = osteoglycin.

The gene correlation analysis of PRDEIG, that is, OGN, found that the top 5 genes positively related to OGN were OMD, SFRP4, TNXB, ecm2 and CCL21; The top 5 negatively correlated genes were GPI, S100A1, ENO1, TNFRSF12A and SLC16A3 (Fig. 4A).

According to the sequence of all relevant genes mentioned above, GSEA enrichment analysis was carried out as the input gene set. The results showed that OGN was significantly enriched in 6 characteristic gene sets, including epithelial mesenchymal transformation (EMT), KRAS signaling up, myogenesis, UV response DN, allograft rejection and apical junction (Fig. 4B). Patients’ prognoses can be affected by OGN effect on the EMT or KRAS signaling pathway, which can help suppress the development of EC.

### 3.5. OGN was associated with immune cell infiltration

**Figure 5.**
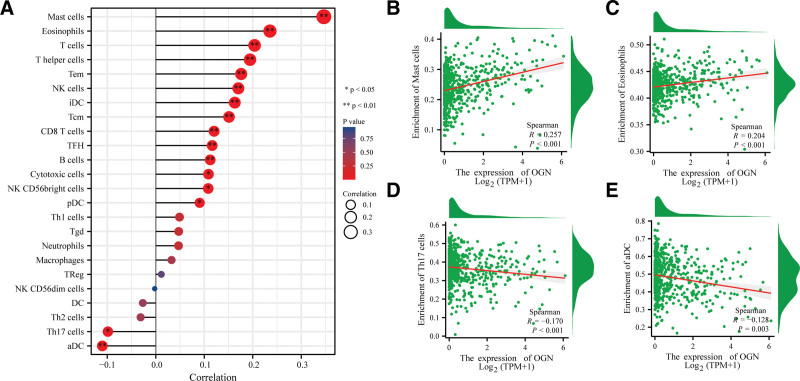
Correlation analysis between OGN and immune cell infiltration. (A) A positive correlation exists between the expression of OGN and 14 types of immune cell infiltration, while a negative correlation exists for 2 types. (B and C) Mast cells and eosinophils are the top 2 immune cells in the positive correlation relationship. (D and E) Infiltration of Th17 and aDC and OGN expression are negatively correlated. OGN = osteoglycin.

The abundance of 24 immune cells in the high and low expression groups of OGN was further compared (Fig. 5A). The results showed that 14 kinds of cells, including mast cells eosinophils and T cells, were significantly higher in the high expression group than in the low expression group, among which mast cells and eosinophils were the top 2 (Fig. 5B and C). In the low expression group of OGN, only Th17 cells and aDC cells increased significantly (Fig. 5D and E). Accordingly, OGN may inhibit the development of EC by altering immune cell function and abundance.

### 3.6. OGN mRNA and protein expression in EC

**Figure 6.**
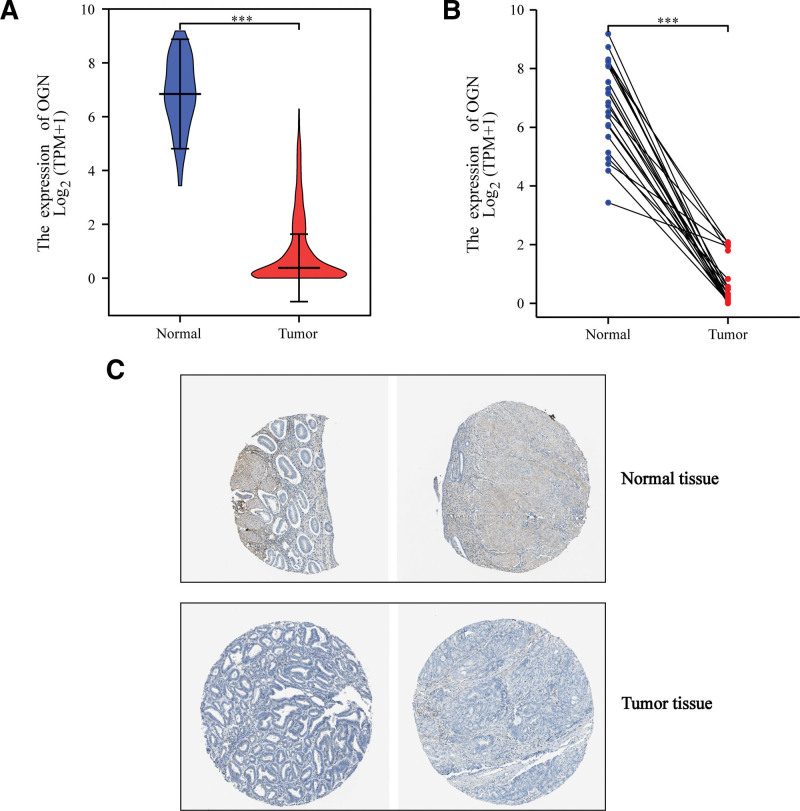
mRNA and protein expression of OGN in EC. (A) A significant reduction in OGN mRNA expression is observed in EC tissues compared to normal tissues. (B) OGN mRNA expression was significantly lower in cancer tissues than in adjacent tissues in paired samples. (C) EC tissue exhibited a significant decrease in the protein level of OGN. EC = endometrial carcinoma, OGN = osteoglycin.

TCGA and THPA databases were used to further analyze the differential expression of OGN mRNA and protein in EC. The results showed that the OGN mRNA expression in EC was significantly lower than that in normal tissues (Fig. 6A). In the paired samples, the down-regulation of OGN expression in cancer tissues was also found (Fig. 6B). THPA database analyzed the expression of OGN protein, and the results were consistent with mRNA. The expression of OGN protein decreased in EC (Fig. 6C).

In light of these findings, OGN plays a significant role in the occurrence, development and prognosis of EC, making it a potential target for immunotherapy of EC.

## 4. Discussion

In recent years, the incidence rate of EC has shown an upward trend.^[13]^ Some patients were found to be in advanced stage, which seriously affected the treatment effect. In addition, routine surgical treatment is not the best choice for some young patients and some patients are too old to tolerate surgery.^[14,15]^ At present, several studies have shown that host immunity and solid tumors have a complex interaction relationship.^[16]^ The existing gene expression database and immune related gene library provide abundant information for exploring tumor immunity.^[17]^ Therefore, it is of great significance to find new immune related biomarkers that can improve the diagnostic value. In this study, IRDEGs in EC were screened through TCGA, GEO and ImmPort databases, and their relationship with clinicopathological parameters and prognosis of EC was further analyzed to obtain PRDEIG, OGN. Through gene expression correlation and GSEA enrichment analysis, we can find the potential functional pathway of OGN, and analyze its association with 24 kinds of immune cells, which provides important information for clarifying the molecular basis of EC occurrence and development and finding its potential immunotherapeutic targets.

By analyzing IRDEGs of EC, we found 4 genes, OGN, LTBP4, CXCL12 and SPP1. Studies have found that OGN can inhibit the proliferation and invasion of tumor cells in breast cancer and bladder cancer.^[18,19]^ In colorectal cancer, OGN inhibits EMT of tumor cells through EGFR/AKT pathway.^[20]^ And it has been identified as a prognosis related gene that affects the tumor immune microenvironment in gastric cancer.^[21]^ LTBP4 was found to affect the proliferation and metastasis of skin melanoma cells,^[22]^ and was also identified as a biomarker related to the prognosis of adrenocortical carcinoma.^[23]^ Research reported that SPP1 can not only affect the tumor dryness of pancreatic cancer,^[24]^ but also mediate the polarization of macrophages and promote the immune escape of lung adenocarcinoma.^[25]^ CXCL12 is an important chemokine, which has been reported in a variety of tumors.^[26]^ However, it has not been found in our study that it can affect the clinical and histological progress of EC and the prognosis of patients. We suspect that CXCL12 should be a tissue-specific chemokine, which cannot play its main function in EC, but this does not explain its important role in other cancers.

The gene correlation analysis of OGN showed that OMD, SFRP4, TNXB, ECM2, and CCL21 ranked top in the positive correlation, while the genes with the top negative correlation were GPI, S100A1, ENO1, TNFRSF12A, and SLA16A3. Among them, the joint role of OMD and OGN in osteoblast biology has been found.^[27,28]^ However, whether these 2 molecules play a common role in cancer is still unclear, which needs to be further explored. SFRP4 and OGN have been identified as the core genes of EC and cancer cell-derived exosome progression,^[29,30]^ which are closely related to the occurrence and progression of EC. The other 8 genes have not been reported together with OGN, which is worthy of further study. GSEA enrichment analysis showed that OGN was mainly enriched in cancer-related signaling pathways such as EMT and KRAS. OGN has been reported in colorectal cancer and cervical cancer to affect the EMT of cancer cells,^[20,31]^ which is consistent with our research results. However, its ability to regulate EMT in EC remains to be explored. In addition, there are no reports that OGN can affect KRAS signaling pathway, which provides clues for subsequent related research.

Recent studies have shown that the immune microenvironment can affect the progression of solid tumors, such as breast cancer, lung cancer and ovarian cancer.^[32]^ The immune microenvironment is considered to be the “fertile soil” for tumor malignant transformation,^[33]^ but there are also many immune factors that can kill tumor cells.^[34]^ A recent study summarized the immune characteristics of a variety of tumors and showed that tumor immune typing and immune cell infiltration characteristics are closely related to the prognosis of patients.^[35]^ However, the study on the immune characteristics of EC is not completely clear.

Our study found that OGN can not only affect the prognosis of EC patients, but also correlate with a variety of immune cells. It suggests that OGN may participate in the regulation of tumor immunity to inhibit the progress of cancer, thus affecting the prognosis of patients. In colorectal cancer, OGN was found to induce VEGF inhibition, thereby enhancing the infiltration of T lymphocytes in the tumor.^[36]^ In gastric cancer, OGN has also been reported to cause differences in immune cell infiltration and immune function between the tumor group and the control group.^[37]^ Research reports that OGN can be considered as a key candidate marker gene for EC,^[29]^ which is basically consistent with our research results. However, it is not clear how it relates to immune infiltration in EC, whether it can affect the progress of EC by regulating immune cell infiltration, and how it can affect the progress of EC, which is worthy of in-depth study.

## 5. Conclusions

In conclusion, 4 IRDEGs, OGN, LTBP4, CXCL12 and SPP1, were found by using TCGA, GEO, and ImmPort databases for differential expression analysis of EC. Analyze their relationship with the prognosis of EC patients, and finally identify a PRDEIG, namely OGN. OGN may exert its function of affecting EMT by affecting the infiltration of tumor immune cells. This provides a new immune target and direction for the research of EC in the future.

## Author contributions

**Data curation:** Yao Lin, Zuhong Teng.

**Formal analysis:** Yao Lin, Zuhong Teng, Guangwei Zhu.

**Funding acquisition:** Yao Lin, Guangwei Zhu.

**Methodology:** Chunlin Lin.

**Project administration:** Songyi Liu, Chunlin Lin, Penghang Lin.

**Software:** Songyi Liu.

**Supervision:** Penghang Lin.
